# A Mouse Model of Autoimmune Cholangitis via Syngeneic Bile Duct Protein Immunization

**DOI:** 10.1038/s41598-017-15661-6

**Published:** 2017-11-10

**Authors:** Wen-Tao Ma, Qing-Zhi Liu, Jing-Bo Yang, Yan-Qing Yang, Zhi-Bin Zhao, Hong-Di Ma, M. Eric Gershwin, Zhe-Xiong Lian

**Affiliations:** 1 Chronic Disease Laboratory, Institutes for Life Sciences and School of Medicine, South China University of Technology, Guangzhou, 510006 China; 2 Liver Immunology Laboratory, Institute of Immunology and The CAS Key Laboratory of Innate Immunity and Chronic Disease, School of Life Sciences, University of Science and Technology of China, Hefei, 230027 China; 3 College of Veterinary Medicine, Northwest Agriculture and Forestry University, Yangling, 712100 China; 4 Division of Rheumatology, Allergy and Clinical Immunology, University of California at Davis School of Medicine, Davis, CA USA; 5 Innovation Center for Cell Signaling Network, Hefei National Laboratory for Physical Sciences at Microscale, Hefei, 230027 China

## Abstract

Primary biliary cholangitis (PBC) is an autoimmune liver disease characterized by the destruction of interlobular biliary ductules, which progressively leads to cholestasis, hepatic fibrosis, cirrhosis, and eventually liver failure. Several mouse models have been used to clarify the pathogenesis of PBC and are generally considered reflective of an autoimmune cholangitis. Most models focus on issues of molecular mimicry between the E2 subunit of the pyruvate dehydrogenase complex (PDC-E2), the major mitochondrial autoantigen of PBC and xenobiotic cross reactive chemicals. None have focused on the classic models of breaking tolerance, namely immunization with self-tissue. Here, we report a novel mouse model of autoimmune cholangitis via immunization with syngeneic bile duct protein (BDP). Our results demonstrate that syngeneic bile duct antigens efficiently break immune tolerance of recipient mice, capturing several key features of PBC, including liver-specific inflammation focused on portal tract areas, increased number and activation state of CD4 and CD8 T cells in the liver and spleen. Furthermore, the germinal center (GC) responses in the spleen were more enhanced in our mouse model. Finally, these mice were 100% positive for anti-mitochondrial antibodies (AMAs). In conclusion, we developed a novel mouse model of PBC that may help to elucidate the detailed mechanism of this complex disease.

## Introduction

Primary biliary cholangitis (PBC) is a prototypical autoimmune liver disease particularly affecting middle-aged women^1–3^. The characteristic histological features of PBC are the destruction of small bile ducts in the portal tracts, biliary epithelial cell apoptosis, and progressive bile duct loss^1,4^. Inflammatory cells aggregate around the injured bile ducts^4^. Despite intense genetic, epigenetic, and immunologic analysis, the etiology of PBC remains enigmatic^5–9^. The bile duct injury results in impaired bile secretion and intrahepatic cholestasis, which further lead to hepatic damage, fibrosis, and eventually cirrhosis or liver failure^1^. Indeed the bile acids themselves may promote inflammation^10^. Serobiochemically, 90–95% of PBC patients are positive for anti-mitochondrial antibodies (AMAs), which predominantly recognize the E2 subunit of the pyruvate dehydrogenase complex (PDC-E2) and, in some cases, the E2 subunit of branched chain 2-oxo-acid dehydrogenase (BCOADC-E2) and 2-oxo-glutarate dehydrogenase (OGDC-E2)^1,11^. These autoantibodies may cross react with both microbial and environmental products^12–14^. Immunologically, the levels of various inflammatory cytokines, such as interleukin (IL)-12 and interferon (IFN)-γ, are increased in the serum of PBC patients^15,16^. It is unclear why the immune attack is predominantly liver-specific, because autoantigens are detected in all nucleated cells. In an effort to investigate the pathogenesis of PBC, several mouse models have been developed. The dominant-negative transforming growth factor-β receptor II (dnTGFβRII) mouse, which was first reported by our group in 2006, has been extensively investigated as a PBC model^17^. This model shares several histological and serological similarities with human PBC, such as bile duct injury, inflammatory cell infiltration in portal tracts, and AMA positivity^17–19^. IL-2Rα^−/−^ mice constitute another model developed by our group, which exhibits the appearance of AMAs, portal inflammation, abnormal T cell activation, and increased inflammatory cytokine levels in the serum^18,20,21^. Furthermore, 2-octynoic acid-BSA (2-OA-BSA)-immunized mice^22^, *N. aromaticivorans*-infected mice^23^, *E. coli*-infected mice^24^, and our recently developed IL-12p40^−/−^CD25^−/−^ mice^25^ have also been used to investigate the pathogenesis of PBC.

Here, we report the establishment of an additional PBC mouse model induced via syngeneic bile duct protein (BDP) immunization that captures several typical features of PBC patients. Our mouse model exhibits a liver-specific inflammation profile, with lymphocyte infiltration in areas of the portal tracts. Furthermore, these mice exhibit more activated T cells in the liver and spleen, and demonstrate enhanced germinal center (GC) responses in the spleen. In particular, increased levels of AMAs, including AMAs against PDC-E2, BCOADC-E2, and OGDC-E2, were identified in the serum of our mouse model. Our results highlight the critical role of bile duct antigens in the pathogenesis of this enigmatic disease.

## Results

### BDP immunization triggers a liver-specific inflammatory response

**Figure 1:**
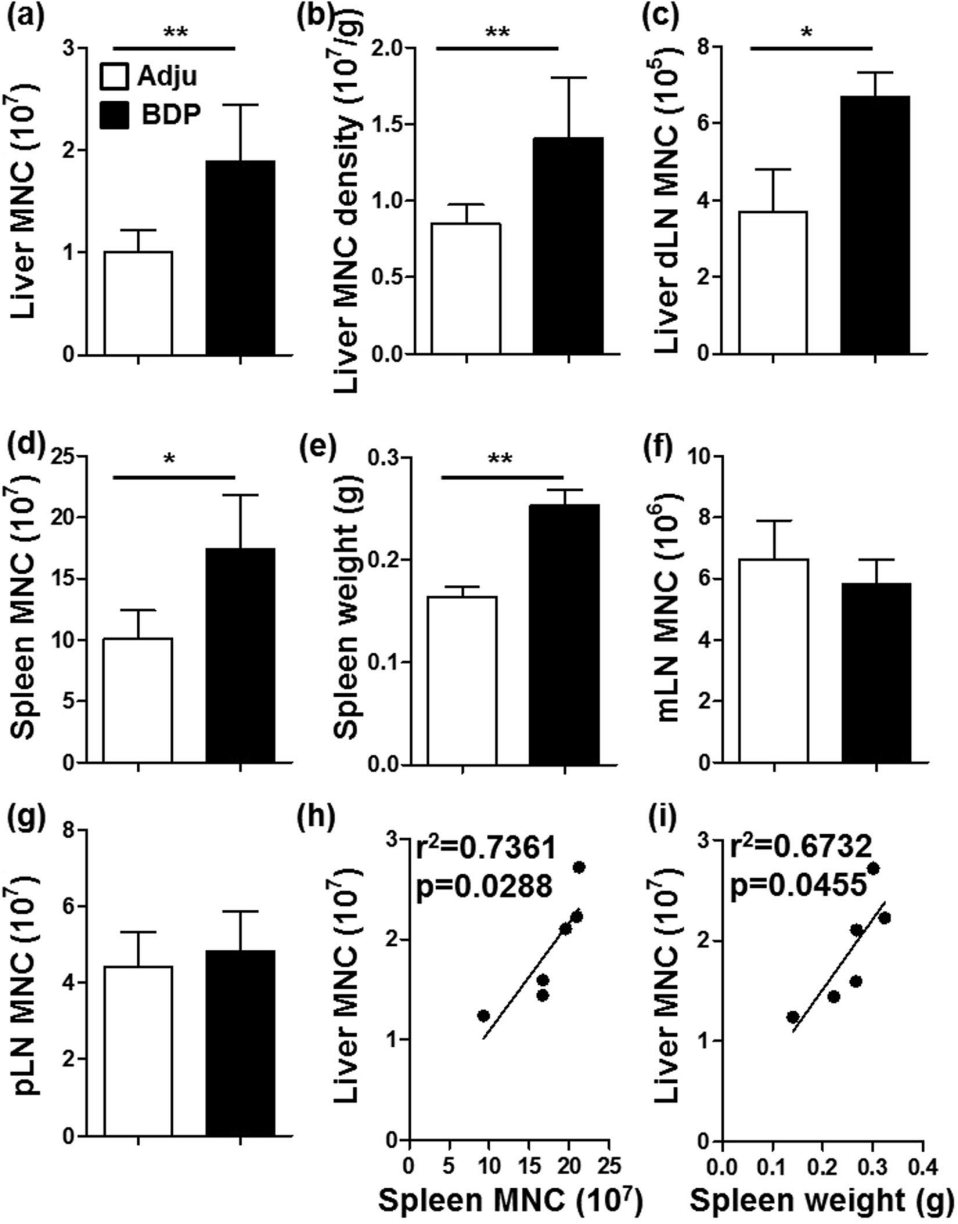
BDP immunization triggered a liver-specific inflammatory response. (**a**–**g**) Total MNC numbers in the liver (**a**), liver dLNs (**c**), spleen (**d**), mLNs (**f**), and pLNs (**g**), as well as the liver MNC density (**b**) and spleen weight (**e**) were compared between adjuvant- (Adju) (n = 6) and BDP-treated groups (n = 6). (**h**,**i**) Linear regression analysis of spleen MNC number and spleen weight versus liver MNC number.

Compared with the mice immunized with adjuvant alone or adjuvant emulsified with small intestinal epithelial cell protein (SIEP), the mice immunized with BDP exhibited a liver-specific inflammation profile (Figs 1a–g and S1a–g), which was evidenced by a significantly increased liver mono-nuclear cell (MNC) number (p = 0.0043, Fig. 1a), increased MNC density in the liver (p = 0.0087, Fig. 1b), and higher MNC number in the liver draining lymph nodes (dLNs) (p = 0.0411, Fig. 1c). We also determined that the spleen MNC number and spleen weight were increased after BDP immunization (p = 0.0152 for the spleen MNC number, p = 0.0048 for the spleen weight, Fig. 1d,e). Moreover, the spleen MNC number and spleen weight were positively correlated with liver MNC number (Fig. 1h,i). In contrast, BDP immunization had no obvious effects on the inflammation of the mesenteric lymph nodes (mLNs) (Fig. 1f) or peripheral lymph nodes (pLNs) (Fig. 1g).

### BDP immunization triggered more severe liver-specific inflammatory infiltration around the bile ducts

**Figure 2:**
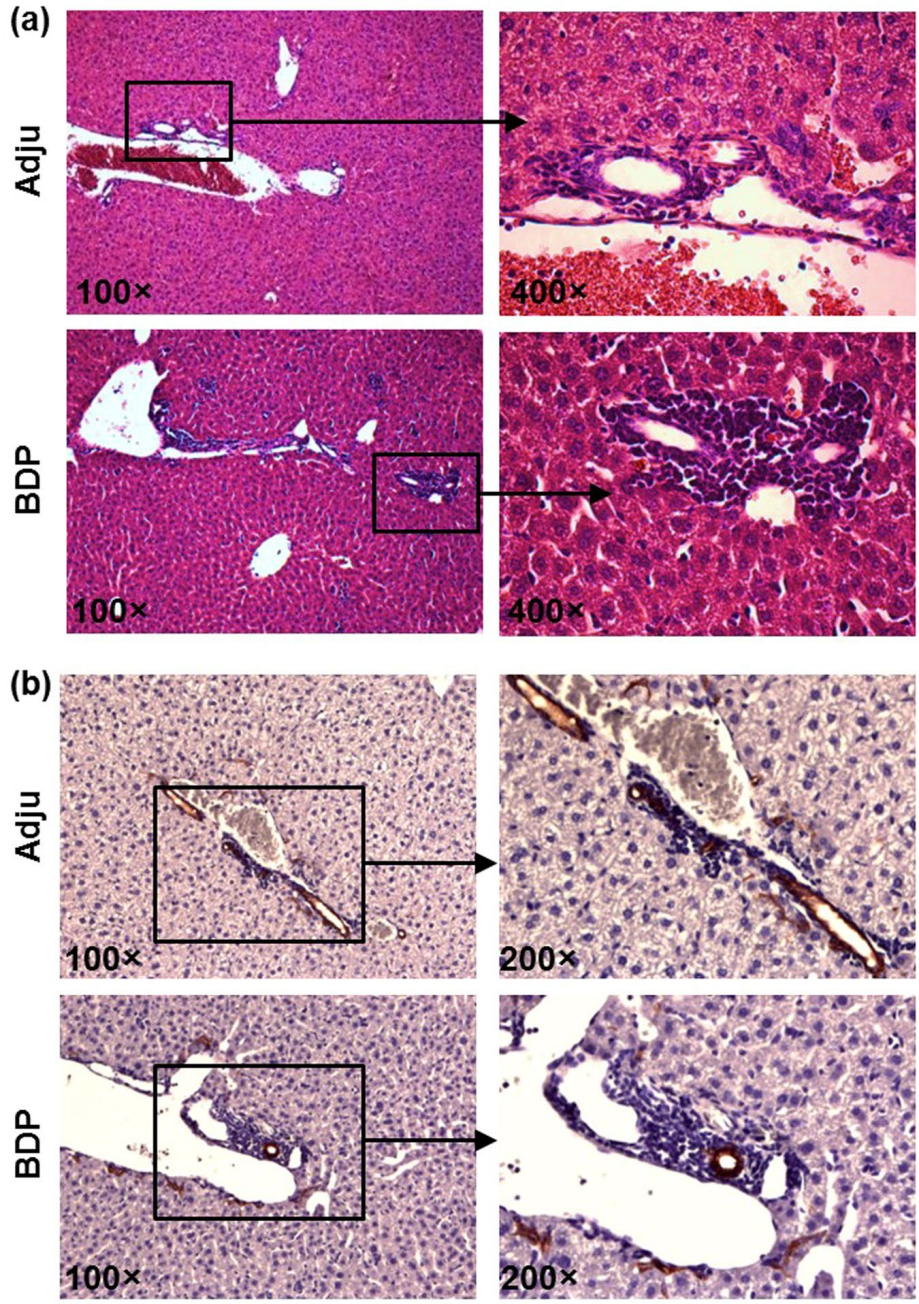
BDP immunization triggered more severe inflammatory infiltration around bile ducts. (**a**) H&E staining of liver sections of adjuvant- (Adju) and BDP-treated mice. (**b**) CK-19 immunohistochemical staining of liver sections from adjuvant- (Adju) and BDP-treated mice.

In contrast with the control adjuvant-treated mice, which exhibited minimal inflammation in the liver, BDP immunization triggered evident inflammation in the liver, and the inflammation areas were mainly focused on the portal tracts (Fig. 2a). In accordance with this finding, immunohistochemical staining of cytokerin-19 (CK-19), which comprised a specific marker for biliary epithelial cells (BECs)^26^, demonstrated that the inflammatory cells were mainly located around CK-19-positive BECs (Fig. 2b). In contrast, there was no inflammation in other tissues, such as the heart, kidney, lung, pancreas, or salivary gland (Fig. S2a–d). However, no granulomas or bile duct damage were observed in both adjuvant- and BDP-treated mice.

### Increases in T and DC cell subsets with a decrease in regulatory T cell (Treg) percentage after BDP immunization

**Figure 3:**
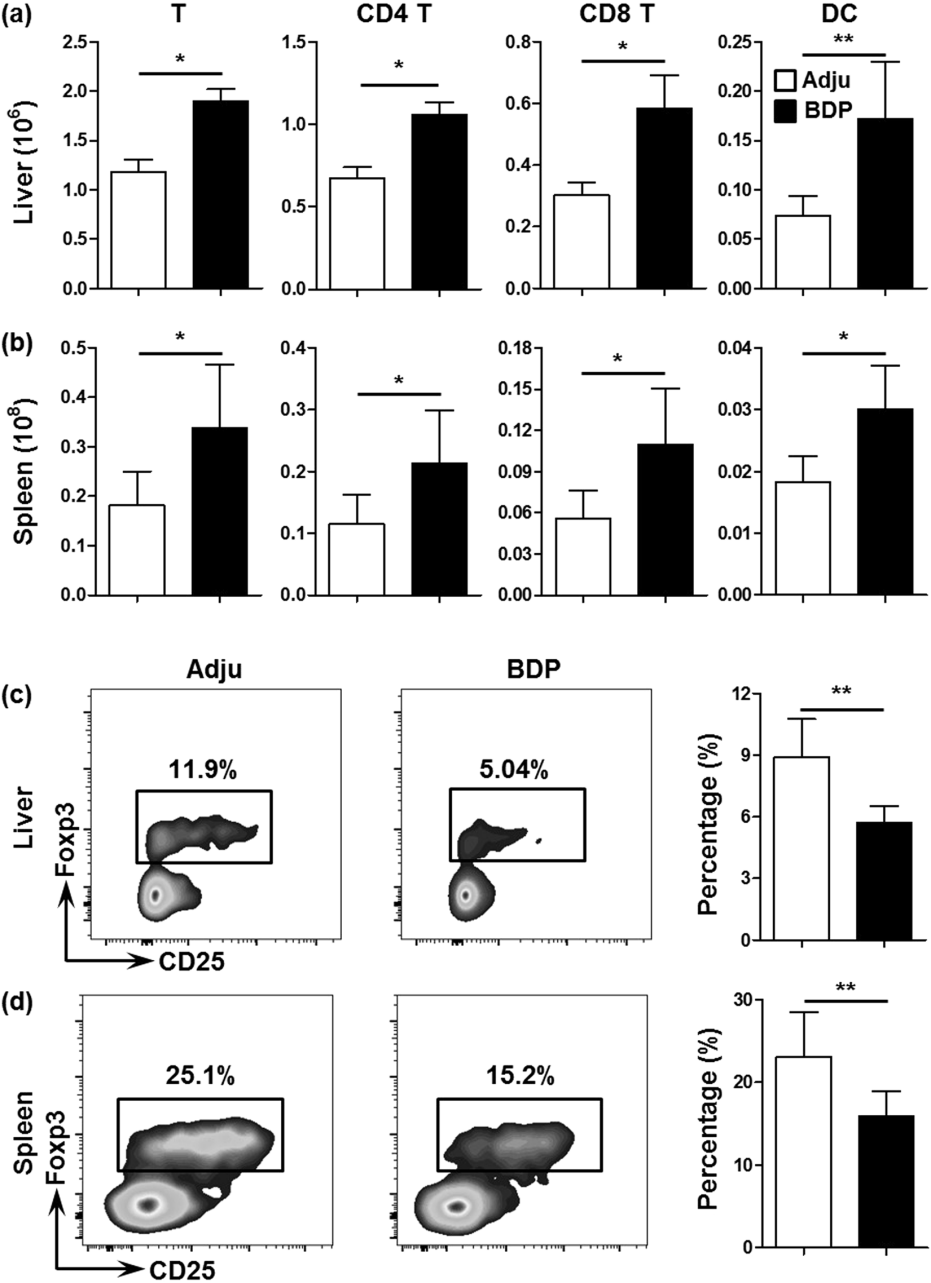
Increases in T and DC cell subsets with a decrease in Treg percentage after BDP immunization. (**a**,**b**) Total numbers of T cells (CD3^+^NK1.1^−^), CD4 T cells (CD3^+^NK1.1^−^CD4^+^CD8^−^), CD8 T cells (CD3^+^NK1.1^−^CD8^+^CD4^−^), and DCs (CD3^−^NK1.1^−^CD11c^high^MHCII^high^) in the liver (**a**) and spleen (**b**) from adjuvant- (Adju) (n = 8) and BDP-treated mice (n = 9). (**c**,**d**) Percentages of CD3^+^CD4^+^NK1.1^−^Foxp3^+^ Treg cells in CD3^+^CD4^+^NK1.1^−^ cells in the liver (**c**) and spleen (**d**) from adjuvant- (Adju) (n = 6) and BDP-treated mice (n = 6) analyzed using flow cytometry.

We further analyzed the specific inflammatory cell subsets in the liver, and determined that the number of T cells, including CD4 and CD8 T cell subsets, significantly increased in the BDP-treated mice compared with the control mice (p = 0.0161 for the liver T cell number, p = 0.0280 for the liver CD4 T cell number, p = 0.0208 for the liver CD8 T cell number, Fig. 3a). In accordance with these findings, in the spleen, the number of total T cells, and CD4 T cell subset as well as CD8 T cell subset, also increased significantly in the BDP-treated mice (p = 0.0260 for the spleen T cell number, p = 0.0152 for the spleen CD4 T cell number, and p = 0.0260 for the spleen CD8 T cell number, Fig. 3b). Interestingly, dendritic cells (DCs), which comprised professional antigen-presenting cells for T cells, were also increased in number in the liver and spleen (p = 0.0048 for the liver DC number and p = 0.0411 for the spleen DC number, Fig. 3a,b). In contrast with the increased T cell number in the liver and spleen in BDP-treated mice, the Treg percentages were significantly decreased in these mice in the liver (p = 0.0087, Fig. 3c) and spleen (p = 0.0022, Fig. 3d) compared with the control mice.

### More activated phenotype of CD4 and CD8 T cells in the liver after BDP immunization

**Figure 4:**
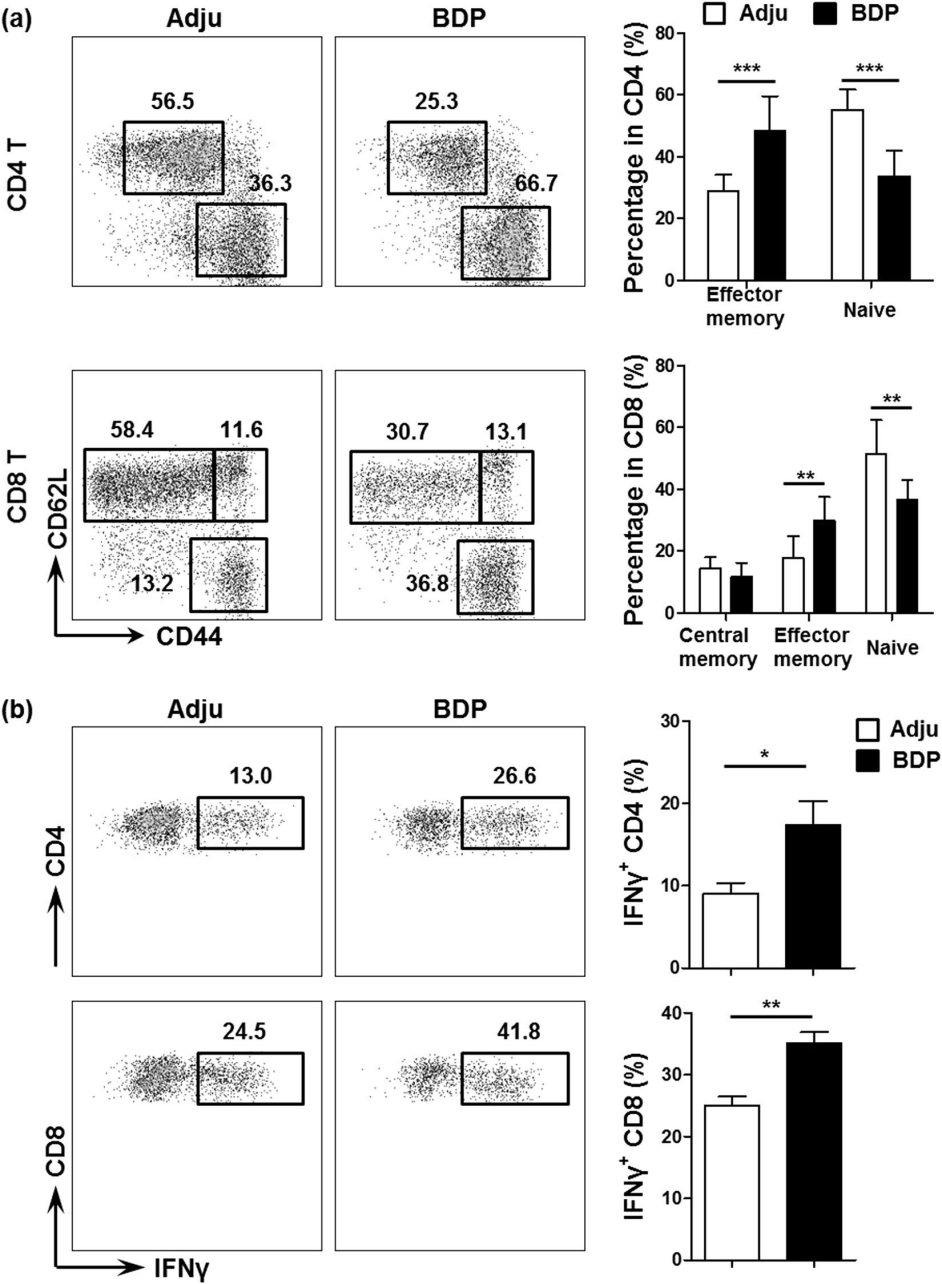
More activated phenotype of CD4 and CD8 T cells in the liver after BDP immunization. (**a**) Flow cytometry analysis of CD44 and CD62L expression levels in liver CD4 (CD3^+^NK1.1^−^CD4^+^CD8^−^) and CD8 T cell (CD3^+^NK1.1^−^CD8^+^CD4^−^) subsets from adjuvant- (Adju) and BDP-treated mice (left panel); percentage analysis of central memory (CD44^high^CD62L^high^), effector memory (CD44^high^CD62L^low^) and naïve T cells (CD44^low^CD62L^high^) (right panel) (n = 9 for Adju and n = 9 for BDP). (**b**) Flow cytometry analysis of liver IFN-γ^+^ CD4 and CD8 T cells (gated from CD3^+^NK1.1^−^) from adjuvant- (Adju) and BDP-treated mice (left panel); percentage analysis of IFN-γ^+^ CD4 and CD8 T cells (right panel) (n = 9 for Adju and n = 9 for BDP).

Based on the increased T cell number results after BDP immunization, we subsequently analyzed the phenotypes of liver and spleen T cells. We determined that in the liver, both the CD4 and CD8 T cell subsets exhibited a more activated phenotype, which was reflected by the increased percentages of memory cells, and the decreased percentages of naïve cells (p = 0.0003 for liver effector memory CD4 T cells, p = 0.0002 for liver naïve CD4 T cells, p = 0.0028 for liver effector memory CD8 T cells, p = 0.0031 for liver naïve CD8 T cells, Fig. 4a). In accordance with these findings, the CD4 and CD8 T cells demonstrated a stronger ability to secrete the inflammatory cytokine IFN-γ (p = 0.0244 for liver IFN-γ^+^ CD4 T cells and p = 0.0038 for liver IFN-γ^+^ CD8 T cells, Fig. 4b). Interestingly, the CD4 T cells in the spleen also exhibited a more enhanced activation state (p = 0.0003 for spleen effector memory CD4 T cells, p = 0.0002 for spleen naïve CD4 T cells). In contrast, CD8 T cell activation profiles in the spleen were not significantly different (Fig. S3a). In addition, IFN-γ secreting ability of both splenic CD4 or CD8 T cells did not show significant differences (Fig. S3b) after BDP immunization.

### Enhanced GC response in the spleen after BDP immunization

**Figure 5:**
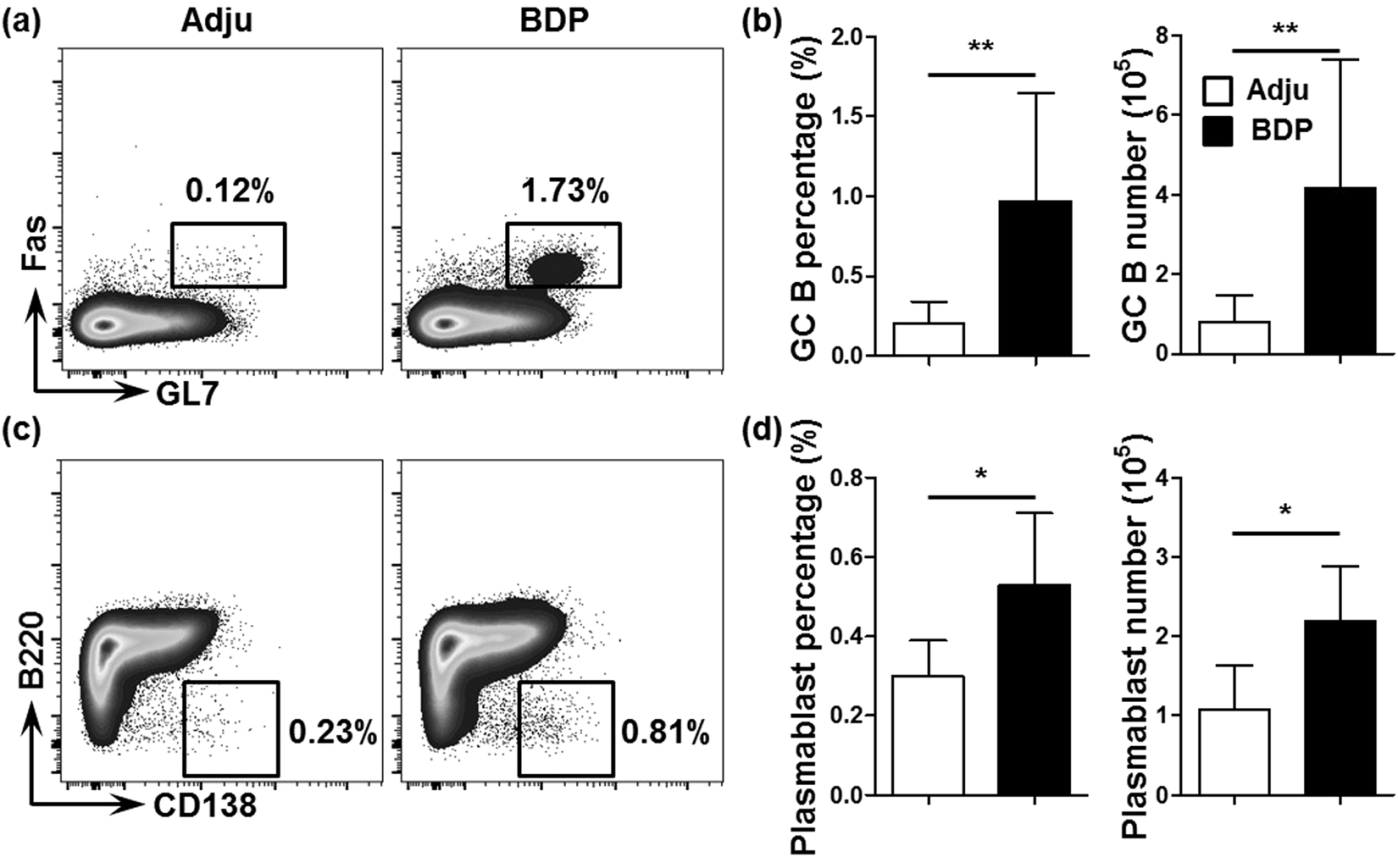
Enhanced GC response in the spleen after BDP immunization. (**a**) GC B cells (CD3^−^CD19^+^IgD^−^Fas^+^GL7^+^) in the spleens of adjuvant- (Adju) and BDP-treated mice analyzed using flow cytometry (left panel). (**b**) Percentage and total number analysis of splenic GC B cells (n = 6 for Adju and n = 6 for BDP). (**c**) Splenic plasmablasts (CD3^−^CD19^+^CD138^hi^B220^lo^) of adjuvant- (Adju) and BDP-treated mice analyzed using flow cytometry. (**d**) Percentage and total number analysis of splenic plasmablasts (n = 6 for Adju and n = 6 for BDP).

We subsequently demonstrated that the GC responses were more enhanced in the BDP-treated mice than in the control mice, and this finding was evidenced by a significantly increased GC B cell percentage and number (p = 0.0043 for GC B-cell percentage, p = 0.0087 for GC B-cell number, Fig. 5a,b), and an increased plasmablast percentage and number (p = 0.0471 for plasmablast cell percentage, and p = 0.0173 for plasmablast cell number, Fig. 5c,d) in the spleens of BDP-treated mice than in the control mice.

### Increased serum AMA levels after BDP immunization

**Figure 6:**
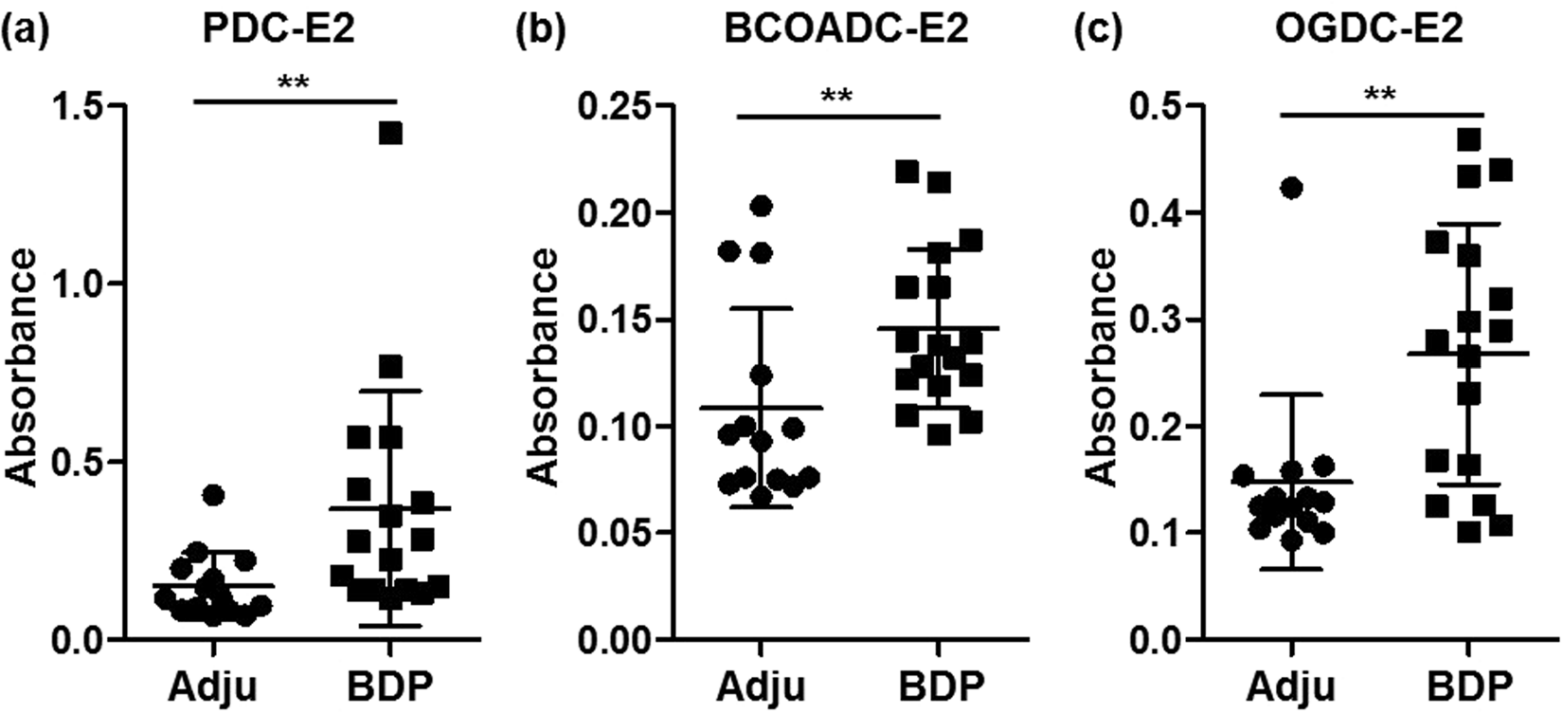
Increased serum AMA levels after BDP immunization. (**a**–**c**) Serum levels of AMAs against recombinant proteins of PDC-E2, BCOADC-E2, and OGDC-E2 analyzed via ELISA at a wave-length of 450 nm.

Considering the increase in the splenic GC responses, we further analyzed the AMA profiles in our mouse model, and the results indicated that BDP immunization triggered significantly elevated levels of serum AMAs against PDC-E2 (p = 0.0043, Fig. 6a), BCOADC-E2 (p = 0.0069, Fig. 6b), and OGDC-E2 (p = 0.0048, Fig. 6c) compared with the control mice.

## Discussion

In PBC patients, a typical histological feature is the progressive destruction of small bile ducts, which may lead to the disappearance of bile ducts and ultimately liver fibrosis and, in some cases, may progress to liver cirrhosis or liver failure. It is known that BECs line the lumen of the intra-hepatic bile ducts and contribute to bile secretion via the release of bicarbonate and water^27^. Interestingly, BECs are also immunologically functional. They express several Toll-like receptors, death receptors, and human leukocyte antigen Class II, which help them to present antigens and secrete inflammatory cytokines. For example, BECs are capable of secreting IL-6, IL-23, and IL-1β to facilitate the differentiation of Th17 cells^28^. Moreover, death receptor 5 (DR5) expression on BECs contributes to common bile-duct ligation-induced cholestasis in mice, and DR5-mediated signaling is also indicated to be involved in the pathogenesis of human cholestatic diseases, such as primary sclerosing cholangitis and PBC^29^. Importantly, apoptotic BECs from PBC patients are positive for autoantigen PDC-E2, and this is reactive with AMA-containing serum, and is capable of activating the immune system to secrete inflammatory cytokines^30,31^. In our mouse model, it is likely that auto-antigens in BDP break down tolerance and initiate the inflammatory responses. The BECs may have, in turn, become the target of the activated immune cells, which aggravated the liver inflammatory response. Moreover, SIEP immunization did not trigger liver inflammation, which was as expected because there was no small intestine-specific antigen in the liver. SIEP immunization also did not trigger inflammation in the intestine, which may indicate that intestinal tolerance is more difficult to break down than that of the liver. We highlighted the theory of tolerance breakdown by the activation of autoimmune lymphocytes via auto-antigens, which was different from genetic mutation-induced autoimmune liver disease in dnTGFβRII mice, IL-2Rα^−/−^ mice and IL-2Rα^−/−^p40^−/−^ mice, and also differed from xenobiotic- or infection-induced autoimmune liver disease in 2-OA-BSA-immunized mice^22^, N. aromaticivorans-infected mice^23^, and E. coli-infected mice^24^.

Several studies suggested that the prevalence of PBC development varies by geographic areas^32^, and environmental toxins, such as volatile aromatic hydrocarbons and trichloroethylene, were significantly associated with a cluster of PBC patients in New York^33^, which suggests the importance of environmental factors for PBC induction^34^. Moreover, the higher risk for the development of PBC in first-degree relatives^35,36^, particularly the higher concordance rate of PBC in monozygotic compared with dizygotic twins^37^, also indicated the critical role of genetic predisposition in this disease. Thus, the pathogenesis of PBC included a combination of environmental factors and genetic predisposition. In the present study, the development of the mouse model was predominantly dependent on bile duct antigens, which in humans may reflect environmental factors that may be mediated by molecular mimicry, and which is similar with the case in 2-OA-BSA-immunized mice^22^, N. aromaticivorans-infected mice^23^, and E. coli-infected mice^24^ on this level. We hypothesize that our mouse model may be modified if genetic predisposition factors are also taken into account. For this purpose, the immunization of BDP in genetically susceptible mice^38,39^ will be performed in further studies.

Other factors related to PBC are gender and age. Some studies have shown organ-specific autoimmune lesions can occur spontaneously in senescent C57BL/6 mice, especially in female mice^40,41^. This is consistent with epidemiological survey of PBC patients^1–3,42,43^. However, in these spontaneous cholangitis mice, the autoimmune lesions first appeared in 6-month-old mice even for female mice. And the previous researches were focused on histologic study and circulating autoantibodies of IgG^40,41^. In our BDP-immunization model, the autoimmune cholangitis was detected in 12-week-old mice. Importantly, we detected high levels of AMAs, which are hallmarks of PBC. Concerning gender factor, we found that male mice was difficult to induce autoimmune cholangitis, which was consistent with the female predominance of PBC. Though we report the new liver-specific PBC mouse model, the mechanism of gender bias for autoimmune diseases is unclear.

A vigorous anti-viral T cell response is required for successful hepatitis viral clearance, whereas persistent viral infection is typically associated with weak T cell activation^44,45^. In addition, T cells are also accounted for the progression of several autoimmune liver diseases^46^. Moreover, T cells participate in other disorders and injuries of the liver, such as ischemia-reperfusion injury^47^, nonalcoholic steatohepatitis^48^, and liver cancers^49^. In our model, T lymphocytes are activated and increased in number, with an increased ability for inflammatory cytokine secretion, which also addressed the critical role of T cells in the pathogenesis of PBC. Compare with other mouse models of PBC, we would see that unbalanced T cell response is a common phenomenon, which appears in the dnTGFβRII mice, the IL-2Rα^−/−^ mice, and the 2-OA-BSA-immunized mice^17,20,25,50–52^.

Tregs are essential for the maintenance of homeostasis and self-tolerance in various organs, including the liver^53^. Treg deficiency or defects will lead to the spontaneous development of various systemic or organ-specific autoimmune diseases, whereas the reconstitution of functional Tregs prevents disease development^54,55^. Tregs play pivotal roles in controlling various liver diseases via the suppression of effector T cell function and cytokine production, and the balance of effector and regulatory T cells always dictates the outcome of various liver diseases^56,57^. Interestingly, in the present mouse model, both the liver and spleen exhibited a reduced Treg percentage, which may be involved in the further activation of T cells. The mechanism of the decreased Treg percentage associated with more severe inflammation is unclear, but this phenomenon is in consistent with other PBC mouse models, such as the dnTGF-βRII mice and IL-2Rα^−/−^ mice^20,58–60^. Importantly, in patients with PBC and even the first relatives of them, Treg deficiency has also been determined^61,62^.

GCs develop in response to antigen stimulation and comprise the sites in which B cells undergo clonal expansion during immune responses. In GCs, rapidly proliferating B cells form foci and undergo somatic hypermutation and affinity-based selection, which eventually produces high-affinity antibodies^63^. GCs are critical for humoral immunity to protect against various infectious diseases; however, aberrant GC responses are associated with numerous autoimmune diseases, such as systemic lupus erythematosus^64^, Sjögren’s syndrome^65^, rheumatoid arthritis^66,67^, autoimmune thyroid disease^68^, and PBC^69,70^. In the present mouse model, enhanced GC responses, as well as increased titers of AMAs were noted. Thus, it is indicated that in our study, bile duct antigens stimulate the generation of abnormal GCs, where autoreactive B cells are generated and expanded, which promotes autoantibody secretion and aggravates liver damage. Future studies should examine gender differences and provide a more robust analysis of innate and adaptive immunity^69,71,72^.

In summary, we report a novel PBC mouse model in which the liver tolerance is broken down by syngeneic BDP immunization, which leads to an abnormal activation of the immune system in the liver and, initiates an autoimmune response resembling that of PBC patients. However, we should state here that no granulomas and bile duct damage were observed in this mouse model, and whether a female bias appears in this model still needs further investigation.

## Methods

### Mice

Wild-type female mice from a C57BL/6 background were purchased from the Shanghai SLAC laboratory animal center (SLAC, Shanghai, China). All mice were maintained in the laboratory animal center of the University of Science and Technology of China in specific pathogen-free conditions. The mice were treated according to the Guide for the Care and Use of Laboratory Animals of the University of Science and Technology of China. All experimental protocols in this study were approved by the Institute of Immunology, University of Science and Technology of China.

### BDP isolation

For BDP isolation, the livers of 8- to 12-week-old mice were perfused *in situ* through the portal vein via collagenase IV (Sigma-Aldrich, St. Louis, Missouri, USA) for 10 min. The liver was then carefully cut and brushed with a soft toothbrush until the whole bile duct tree was clearly visualized. The bile duct tree was placed in collagenase IV (Sigma-Aldrich) and shaken for 10 min to separate the adhering hepatocytes. After washing in phosphate-buffered saline (PBS) for 3 times, the bile duct tree was thoroughly cut and homogenized in PBS via an ultrasonic disruptor (Xinzhi, Ningbo, China). After centrifugation at 12,000 × *g* and 4 °C for 5 min, the supernatant was collected, and the protein concentration was determined using a BCA protein assay kit (Thermo Fisher Scientific, Waltham, MA, USA).

### BDP immunization protocol

Seven-or eight-week-old mice (SLAC, Shanghai, China) were used for BDP immunization. BDP at a concentration of 4000 µg/ml was emulsified with an equal volume of complete Freund’s adjuvant (CFA) purchased from Sigma-Aldrich. Following successful emulsification, 200 µl BDP-CFA emulsion were subcutaneously injected at multiple points on the back of a mouse. This BDP-CFA emulsion was used for 3 treatments. BDP was subsequently emulsified with incomplete Freund’s adjuvant (IFA) purchased from Sigma-Aldrich. This method and dose were the same as BDP-CFA. The BDP-IFA emulsion was used only in the last treatment. One week after the BDP-IFA treatment, the mice were sacrificed and analyzed. Control mice were treated via the emulsion of CFA or IFA with an equal volume of 0.9% NaCl solution or SIEP, and the injection dose and volume were the same as the experimental group (Fig. S4).

### SIEP isolation

For SIEP isolation, the small intestines were cut off and opened to clear out the faeces with PBS. The small intestines were subsequently cut into 1-cm fragments in 30 mM EDTA and shaken at 37 °C for 10 min. The supernatants were transferred to cold PBS and centrifuged at 500 × *g* for 5 min. The pellets that contained intestinal crypt cells were subsequently collected and homogenized in PBS via an ultrasonic disruptor (Xinzhi). After centrifuging at 12000 × *g* and 4 °C for 5 min, the supernatant was collected, and the protein concentration was determined using a BCA protein assay kit (Thermo Fisher Scientific).

### Flow cytometry

MNCs from the liver, spleen, and mLNs were isolated as previously described^25^. The method for MNC isolation from dLNs and pLNs was the same as that for mLNs.

For staining cell surface markers, isolated MNCs were treated with anti-CD16/32 (Biolegend, San Diego, CA, USA) for 15 min, and subsequently incubated for 20 min with fluorescent antibodies. The fluorescent antibodies included FITC-conjugated anti-I-A/I-E (M5/114.15.2), anti-CD3 (17A2); PE-conjugated anti-CD25 (PC61); PerCP/Cy5.5-conjugated anti-CD11c (N418), anti-GL7 (GL7); PE/Cy7-conjugated anti-NK1.1 (PK136), anti-CD19 (6D5); APC/Cy7-conjugated anti-CD4 (GK1.5), anti-IgD (11–26 c.2a), anti-CD4 (GK1.5); and Pacific Blue-conjugated anti-CD3 (17A2), anti-NK1.1 (PK136), anti-CD45R (RA3-6B2), which were all purchased from Biolegend. PE-conjugated anti-CD138 (281-2) and V500-conjugated anti-CD8α (53-6.7) were purchased from BD Biosciences (Franklin Lakes, New Jersey, USA). Alexa 488-conjugated anti-CD95 (15A7) was purchased from eBioscience (San Diego, CA, USA).

For Foxp3 staining, a Foxp3 detection kit (eBioscience) was used according to the manufacturer’s instructions. Alexa 647-conjugated Foxp3 (150D, Biolegend) was used for intracellular staining.

For IFN-γ staining, MNCs were suspended in RPMI 1640 medium (Thermo Fisher Scientific) with 10% fetal bovine serum (Millipore, Billerica, MA, USA) and stimulated with a Cell Stimulation Cocktail (eBioscience) at 37 °C for 4 h. The MNCs were subsequently fixed with a fixation/permeabilization buffer kit from BD Biosciences according to the manufacturer’s instructions. PE or PE/Cy7-conjugated anti-IFN-γ (XMG1.2) antibody (Biolegend) was used for intracellular staining.

A FACSVerse flow cytometer (BD Biosciences) was used to collect the flow cytometry data, and data analysis was performed using Flowjo software (Tree Star, Ashland, OR, USA).

### Histology

Fresh tissues were fixed in 4% paraformaldehyde for 48 h, and subsequently embedded with paraffin. The samples were cut into 4-μm sections and deparaffinated at 60 °C for 40 min. The sections were subsequently stained with H&E and viewed under a light microscope as previously reported^58^. To specify BECs, immunohistochemical staining of cytokerin-19 was assessed by staining with anti-cytokeratin 19 (Abcam, Cambridge, UK) and visualized with a ChemMate EnVision Detection Kit (Dako, Produktionsvej, Denmark)^73^.

### Enzyme-linked immunosorbent assay (ELISA) for analysis of AMAs

Recombinant PDC-E2, BCOADC-E2, and OGDC-E2 were coated overnight at 4 °C at a concentration of 15 μg/ml in a 96-well-plate. The plates were subsequently blocked with 3% skimmed milk for 1 h. Serum diluted 1/20 was added to the plates for 1 h. Anti-mouse IgG peroxidase (Sigma-Aldrich) was subsequently added to the plates for 1 h. Finally, a TMB Substract Reagent Set (BD Biosciences) was added for 10 min. Following the addition of stop solutions (BD Biosciences), the optical density was read at 450 nm.

### Statistical analysis

All data are representative of at least 3 replicates and are presented as the mean ± standard deviation (SD). Mann-Whitney U-test was used to analyze the differences between two groups. The confidence interval is 95%. The differences were significant if the p value was less than 0.05: *p < 0.05, **p < 0.01, and ***p < 0.001. All data are analyzed using a GraphPad Prism software (GraphPad Software, Inc., San Diego, CA, USA).

## Electronic supplementary material

Supplementary Figure legends
