# Supplementary material for: A Mouse Model of Autoimmune Cholangitis via Syngeneic Bile Duct Protein Immunization
Source: Sci Rep. 2017 Nov 10;7:15246. doi: 10.1038/s41598-017-15661-6 (PMC5681628; doi:10.1038/s41598-017-15661-6)

A Mouse Model of Autoimmune Cholangitis via Syngeneic Bile Duct Protein Immunization

Wen-Tao Ma[1,2,3#], Qing-Zhi Liu[1,2#], Jing-Bo Yang[2], Yan-Qing Yang[2], Zhi-Bin Zhao[1,2], Hong-Di Ma[2], M. Eric Gershwin[4] and Zhe-Xiong Lian[1,2,5*]

[1]Chronic Disease Laboratory, Institutes for Life Sciences and School of Medicine, South China University of Technology, Guangzhou 510006, China;

[2]Liver Immunology Laboratory, Institute of Immunology and The CAS Key Laboratory of Innate Immunity and Chronic Disease, School of Life Sciences, University of Science and Technology of China, Hefei 230027, China;

[3]College of Veterinary Medicine, Northwest Agriculture and Forestry University, Yangling 712100, China;

[4]Division of Rheumatology, Allergy and Clinical Immunology, University of California at Davis School of Medicine, Davis, CA, USA;

[5]Innovation Center for Cell Signaling Network, Hefei National Laboratory for Physical Sciences at Microscale, Hefei 230027, China.

[#]These authors contributed equally to this work.

.

Supplementary Figure legends

Fig. S1 SIEP immunization did not trigger liver-specific inflammation.

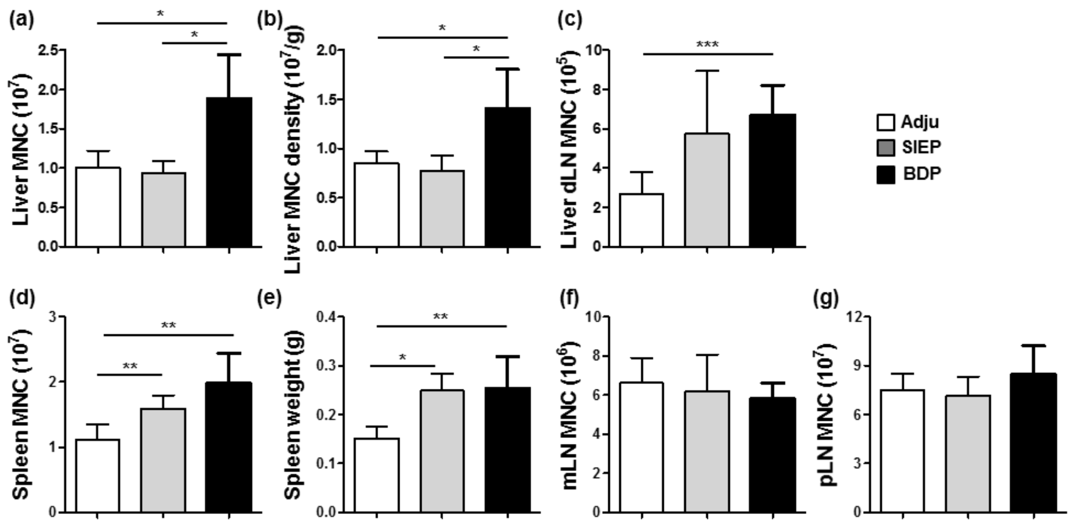

(a-g) Total mono-nuclear cell (MNC) number in the liver (a), liver draining lymph nodes (dLN) (c), spleen (d), mesenteric lymph nodes (mLN) (f), and peripheral lymph nodes (pLN) (g), as well as liver MNC density (b) and spleen weight (e), were compared between adjuvant- (Adju), SIEP-, and BDP-treated mice (n=6 for Adju, n=5 for SIEP, and n=6 for BDP).

Fig. S2 Histological profiles of various tissues of adjuvant- (Adju) and BDP-treated mice.

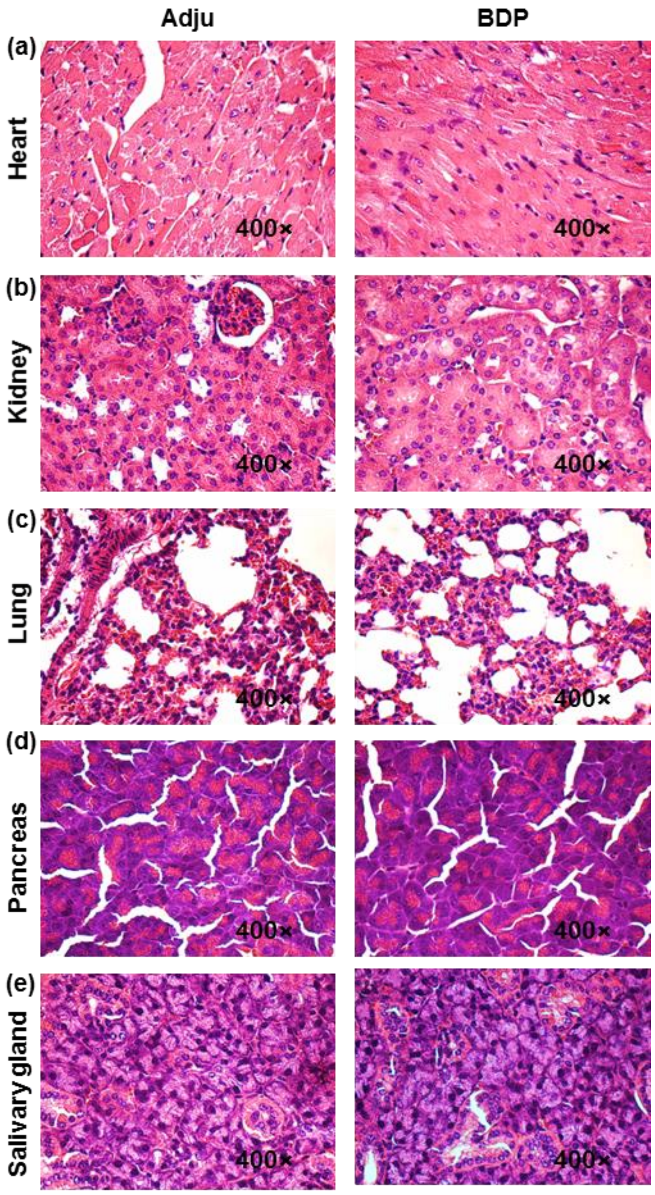

(a-e) HE staining of tissues including the heart, kidney, lung, pancreas, and salivary gland from adjuvant- (Adju) and BDP-treated mice.

Fig. S3 More activated phenotype of CD4 and CD8 T cells in the spleen after BDP immunization.

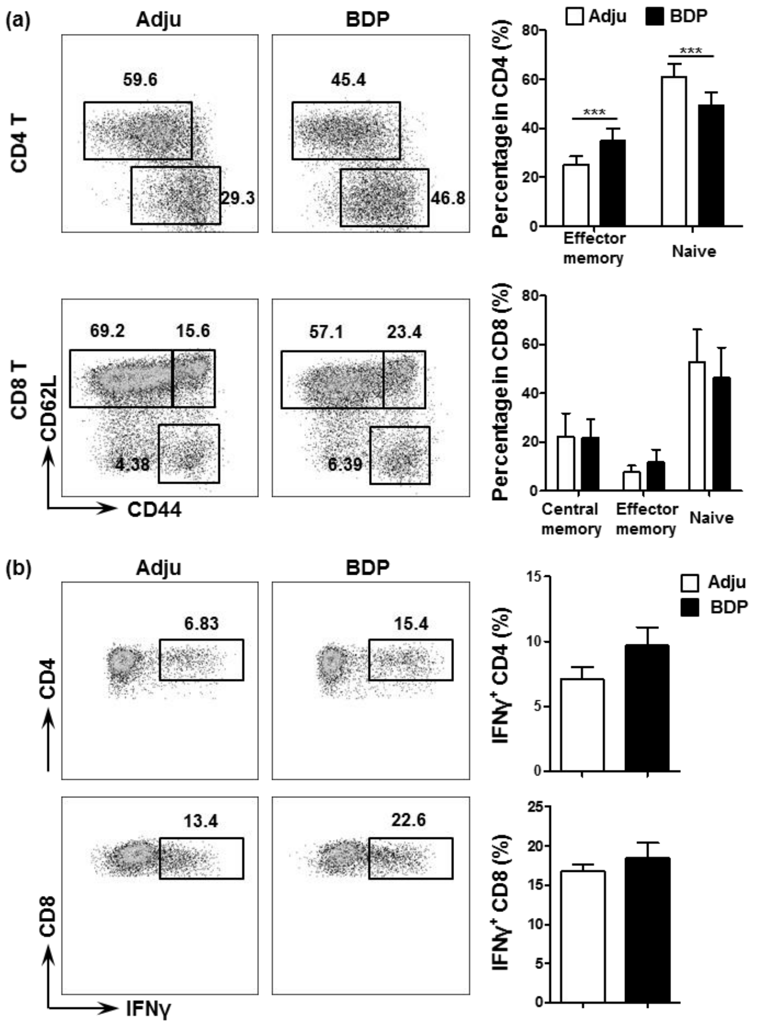

(a) Flow cytometry analysis of CD44 and CD62L expression levels in splenic CD4 ($CD3^+NK1.1^-CD4^+CD8^-$) and CD8 T cell ($CD3^+NK1.1^-CD8^+CD4^-$) subsets from adjuvant- (Adju) and BDP-treated mice (left panel). Percentage analysis of central memory ($CD44^{high}CD62L^{high}$), effector memory ($CD44^{high}CD62L^{low}$) and naïve T cells ($CD44^{low}CD62L^{high}$) (right panel) (n=9 for Adju and n=9 for BDP). (b) Flow cytometry analysis of splenic IFN-γ^+ CD4 and CD8 T cells (gated from $CD3^+NK1.1^-$) from adjuvant- (Adju) and BDP-treated mice (left panel); and

the percentage analysis of IFN-γ^+ CD4 and CD8 T cells (right panel) (n=9 for Adju and n=9 for BDP).

Fig. S4 Protocol for BDP immunization (see BDP immunization protocol in detail in section of Methods).

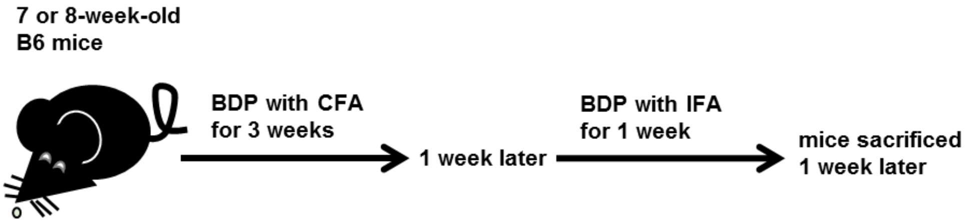

Supplement: Supplementary file 1 — Supplementary Figure legends [file 41598_2017_15661_MOESM1_ESM.pdf]
